# Microbial Musings – June 2020

**DOI:** 10.1099/mic.0.000951

**Published:** 2020-06-30

**Authors:** Gavin H. Thomas

**Affiliations:** ^1^​ University of York York, UK

As we move into the summer of this *annus horribilis*, we can see the pandemic at different stages across the globe. In some parts of Europe there are strong moves, rightly or wrongly, to return to some sense of normality, while in other places, particularly in South America at the moment, cases are still on the rise. In the UK an incredible study led by many members of the UK microbiology community provided insight at unprecedented levels into how the disease was introduced into the country, using genomics to track over 1500 independent entry events of the virus in the key month before lockdown. This tour de force in molecular epidemiology was undertaken by the COVID-19 Genomics UK consortium (Cog-UK), which included many Microbiology Society members and meant another Radio 4 interview for the ubiquitous Nick Loman (@pathogenomenick) from the University of Birmingham.

This month we start the issue with an interesting review about gas vesicles in bacteria and archaea by Amy Hill and George Salmond from the University of Cambridge [1]. These intracellular protein-lined compartments are formed and matured through processes that are not fully understood. Their key feature is that they are totally impermeable to water, while allowing dissolved gas to diffuse in to create gas-filled vesicles. The primary function in cyanobacteria is thought to be in regulating buoyancy of the bacteria in the water column. The review covers their structure and function and also considers their use in diagnostics and biotechnology as other proteins can be fused to one of the vesicle surface proteins, GvpC, to present vaccines. Remarkably, these vesicles can also be expressed and will form in eukaryotic cells and can be used as contrast agents detectable by magnetic resonance imaging (MRI).

The first time I had to think about gas vesicles was when I was being interviewed to read microbiology at the University of Bristol in the early 1990s. The lovely Alan Hedges, one of the admissions tutors, asked me what I thought the function of gas vesicles was in marine bacteria. I had no idea, but remember saying something about how they might be products of endocytosis and involved in nutrient acquisition. Despite being totally wrong, I got a place and I have always assumed that I got some credit for lateral thinking! Clearly the question was a hint about the research strengths of the department, as two world leaders in the characterization of gas vesicles, Tony Walsby and Paul Hayes, were members of the teaching staff. Satisfyingly, Walsby’s pioneering early work on gas vesicles was published in this journal [2, 3]. As a young undergraduate, Walsby always appeared to me to have the dream microbiology career, spending lots of his year in exotic places discovering new microbes. Just before I arrived this activity peaked with his amazing discovery of square archaea [4], later named *Haloquadratum walsbyi *, after their discoverer.

Our second highlighted paper this month brings me closer to some of our current projects concerning industrial biotechnology and bioenergy and the solventogenic clostridia (@Detox_Project). These Gram-positive spore-forming bacteria are metabolically unusual in that they undergo a two-stage fermentation of sugars to first produce acids (acidogenesis) and then, when the pH drops, change their physiology to a second stage, solventogenesis, when they reassimilate the acids and produce neutral solvents such as butanol. This is a last gasp that just precedes their irreversible journey on the road to sporulation [5] . Despite these bacteria being used since the First World War to produce acetone for cordite manufacturing, the fundamental physiology of these growth transitions are still poorly understood. The group of Klaus Winzer, working within the larger Synthetic Biology Research Centre (SBRC) at the University of Nottingham (@SbrcNottingham), has published an important new paper offering some insight into this transition [6]. Their study analyses and characterizes some of the multitude of potential quorum-sensing systems made by the solvent producer *Clostridium acetobutylicum *. They discover eight potential systems of the RRNPP type in the genome and by sequentially inactivating them they discover that one particular system, QsrB, is important for the acid–solvent transition and the amount of butanol produced. The *qsrB* mutant strain has increased solvent formation and overexpressing the system causes the reverse phenotype. Significantly, this can be reversed by adding a synthetic version of the predicted signalling peptide, which is then, presumably, taken up and bound by the regulator protein, QsrB. While the targets for regulation through this system are not yet established, the paper presents clear evidence that manipulating quorum sensing could have potential applications in increasing butanol production in bacteria. The unexpected complexity and potential importance of quorum sensing in *C. acetobutylicum* reminded me of a nice paper from 2017, where the same bacterium was discovered to make polyketide-type signalling molecules that are important for the progression through the late steps of the fermentation into sporulation, again suggesting that a lot more basic physiology needs to be understood to realize the maximum potential of these microbes [7].

Sticking with biotechnology, we have a nice methods-based paper in this issue about using synthetic biology to create multigene insertions in the algal chloroplast genomes. This is the work of Marco Larrea-Alvarez and *Microbiology* editor Saul Purton (@AlgaeProfessor) at UCL, London, UK and aims to improve genetic methodologies for *Chlamydomonas reinhardtii*, which can be considered to be a potential eukaryotic cell factory. As these photosynthetic microbes contain chloroplasts, which have their own genomes, called the plastome, they can be manipulated to introduce foreign genes for overproduction and also to introduce enzymes that can alter chloroplast metabolism to produce new chemicals [8]. In this work the authors assemble multigene constructs and are able to move them in a single event onto the chloroplast genome, being able to detect synthesis of the products of all three genes they introduced at once [9]. While promising, they also note that care must be taken in designing the regulatory elements for each separate transcriptional unit and they see recombination between 3′UTRs when they are very similar. To address they aim to use John Heap’s (@johntheap) newly published start–stop assembly method [10] to quickly assembly non-homologous regulatory elements to overcome their stability problem.

Our last two highlighted papers focus on peptidoglycan and antibiotics. The first takes us back to the genus *Clostridium* as we turn to a pathogenic species *C. difficile* – although formally this has been reclassified as *Clostridioides difficile *, after a brief sojourn as *Peptoclostridium difficile* [11]. One mechanism to confer resistance to the antibiotic vancomycin, first discovered in other Gram-positive bacteria, is to alter part of its target site through amidation of the *meso*-diaminopimelic acid in the peptide cross-bridge [12]. The paper in this issue looks at the function of this process in *C. difficile *, which the authors, led by Thomas Candela and colleagues at the Université Paris-Saclay, France, demonstrate is present in peptidoglycan isolated from *C. difficile* and which requires the *asnB* gene product [13]. Expression of this gene also appears to be induced by the presence of sub-inhibitory concentrations of vancomycin, through an unknown mechanism. Further, the modifications only confer a small phenotype relating to vancomycin resistance, so possibly these modifications have other physiological functions that remain to be elucidated. Our second peptidoglycan paper concerns the characterization of an additional dd-carboxypeptidase from the bacterium *Mycobacterium smegmatis* that is involved in remodelling of the cell wall. The paper from the group of Anindya Ghosh, at the Indian Institute of Technology in Kharagpur, India, is a continuation of a story they published in *Microbiology* in 2015 that characterized the MSMEG_2433 protein [14]. In this paper they find that the adjacent gene product MSMEG_2432 has an essentially identical function as a DD-carboxypeptidase that is able to remove the terminal d-alanine residue from the murein pentapeptide [15]. Disruption of this gene pair changes the shape and length in *M. smegmatis* [16], although individual disruptions appear to have no phenotype, suggesting that there is some redundancy of function in these enzymes, and in fact, *M. smegmatis* appears to have four enzymes of this type [17]. A nice tool the authors used in this paper is an *Escherichia coli* strain, CS703-1, which has deletions of seven different genes involved in peptidoglycan metabolism and looks suitably sickly down a microscope [18, 19]. They show nicely that MSMEG_2432 can rescue this phenotype, but a strain containing the same gene with a point mutation that alters a likely catalytic residue has a much poorer phenotype.

That is everything for this month. Next month I will be flagging our first *Microbiology* community-led review article, which is something we are hoping to develop more of in 2021. Look out for July’s *Musings* to find out about this and other developments for the journal.
